# Personality, eating behaviour, and body weight: results from the population study of women in Gothenburg 2016/17

**DOI:** 10.1038/s41366-025-01764-y

**Published:** 2025-04-04

**Authors:** Lena Farhan, Dominique Hange, Tore Hällström, Cecilia Björkelund, Lauren Lissner, Lisbeth Stahre, Kirsten Mehlig

**Affiliations:** 1 School of Public Health and Community Medicine, Institute of Medicine, Sahlgrenska Academy, University of Gothenburg, Gothenburg, Sweden; 2 Research, Education, Development & Innovation, Primary Health Care, Region Västra Götaland, Gothenburg, Sweden; 3 Neuropsychiatric Epidemiology Unit, Department of Psychiatry and Neurochemistry, Institute of Neuroscience and Physiology, Sahlgrenska Academy, University of Gothenburg, Gothenburg, Sweden; 4 Department of Clinical Neuroscience, Section for Psychiatry, Huddinge, Karolinska Institute, Stockholm, Sweden

**Keywords:** Epidemiology, Risk factors

## Abstract

**Aims:**

The aim was to investigate the cross-sectional associations between personality traits, psychogenic needs and eating behaviour, and to describe the extent to which personality influences the association between eating behaviour and weight status.

**Methods:**

In 2016/17, a population-based sample of 573 women in Gothenburg, Sweden aged either 38 or 50 participated in a health examination. They completed the Three-Factor Eating Questionnaire, measuring uncontrolled eating, emotional eating and cognitive restraint on a scale of zero to 100. Scores higher than 50 defined excessive eating behaviour. The Cesarec-Marke Personality Schedule was used to measure psychogenic needs, characterised by pursuits and objectives that define personality and influence actions. Extraversion and neuroticism were assessed using the Eysenck-Personality Inventory. Regression models for excessive eating behaviour and for logarithmic body mass index (BMI) as a function of standardised personality scores were adjusted for sociodemographic, lifestyle and health factors.

**Results:**

A higher need to defend one’s status (DST) was positively associated with excessive uncontrolled eating, odds ratio (OR) = 1.44, 95% confidence interval = (1.11, 1.86) per standard deviation (SD) of DST. The need to defend one’s status was more strongly associated with excessive emotional eating, OR = 1.61 (1.18, 2.20) than neuroticism, OR = 1.45 (1.06, 1.97), in a mutually adjusted model. Needs for achievement and autonomy were associated with excessive cognitive restraint, OR = 1.39 (1.09, 1.76) and 0.78 (0.62, 0.97), respectively. Excessive emotional eating was associated with 5.3 (1.1, 9.6) % higher values of BMI when adjusted for the need of DST, which was associated with −2.7 (−4.1, −1.3) % lower BMI per SD.

**Conclusions:**

Psychogenic needs were more closely associated with eating behaviour than personality traits. A lower need to defend one’s status and excessive emotional eating were independently associated with higher BMI, suggesting different pathways to obesity and treatment strategies.

## Introduction

The global prevalence of obesity remains high, and further research into the psychological underpinnings of eating behaviour is essential to develop targeted strategies for weight loss and obesity management [1]. The Three-Factor Eating Questionnaire (TFEQ) is a validated instrument for measuring eating behaviour in the general population and defines three principal domains of adverse eating behaviours: uncontrolled eating, which is characterised by the tendency to lose control over food intake and overeat, emotional eating, which describes the inclination to eat as a way of coping with dysphoric states, and cognitive restraint, which is defined as consciously restricting food intake regardless of feelings of hunger [2–5]. We have recently shown that emotional eating was the strongest correlate of various measures of adiposity in a population-based sample of women in Sweden [6]. Previous studies on the relationship between personality and eating behaviour have mainly focussed on the Big Five personality traits, of which neuroticism has the strongest, positive association with emotional eating and eating disorders [7]. Higher levels of neuroticism or extraversion and lower levels of conscientiousness have also been linked to higher BMI [8]. Knowledge about the association between personality traits and eating behaviour may inform the psychiatric treatment of overeating and obesity. However, the Big Five personality traits are considered to be innate characteristics that remain stable throughout life [9], limiting the possibility to modify these aspects of unhealthy weight status.

Psychogenic needs present an alternative model for describing personality that are more closely linked to the motives that lead to certain behaviours and that may be more amenable to treatment and change. Psychogenic needs are assessed using the Cesarec-Marke Personality Schedule (CMPS), which is the Swedish version of the Edwards Personal Preference Scale [10]. According to Murray’s theory of personality two sets of needs are defined in humans [11]. The primary needs are centred upon basic biological requirements such as food, water and sex. The secondary or ‘psychogenic’ needs are characterised by goals and pursuits such as affiliation, nurturance, and dominance. Secular changes in psychogenic needs were observed in cohorts of 38- and 50-year-old women, who participated in the 1968 and 2004 examinations of the Population Study of Women in Gothenburg (PSWG), with higher scores for the needs of achievement, dominance, aggression and exhibition in the more recent cohort [12]. This pattern was also observed in a study of 75-year-old men and women examined in 1976 and 2005. The latter study also showed that the gender gap in the values for the need of achievement, the desire to accomplish something challenging, had decreased [13]. This could be due to societal change, where women increasingly occupy a more dominant position. Consequently, one might assume that psychogenic needs are also changeable in the individual, but longitudinal studies of psychogenic needs are lacking.

To our knowledge, the relationship between psychogenic needs and eating behaviour as well as their combined association with body weight has not been investigated before. Hence, we assessed the association between psychogenic needs and eating behaviour measured by the Three-Factor Eating Questionnaire in 38- and 50-year-old women, who participated in the 2016 PSWG survey. Furthermore, we examined whether previously observed associations between eating behaviour and obesity measures [6] were influenced by further adjustment for psychogenic needs. Participants also responded to the Eysenck personality inventory assessing extraversion and neuroticism [14, 15], allowing us to compare the associations of psychogenic needs and personality traits with eating behaviours and body weight.

## Materials and methods

### Study population and design

A population-based sample of 573 women aged 38 (*n* = 263) and 50 (*n* = 310) was retrieved from the Prospective Study of Women in Gothenburg, using the data from the most recent examination in 2016/17. Participation rates were 63% and 73% among 38- and 50-year-old women respectively [16]. The original cohort was established in 1968 with focus on menopause and cardiovascular disease, which was the reason for choosing the age strata 38, 46, 50, 54, and 60 [17]. Subsequent examinations were performed every 12^th^ year and included new samples of 38- and 50-year-old women, to allow for the investigation of secular trends [12]. Hence, the present study is cross-sectional in nature.

### Assessment of eating behaviour

Participants’ eating behaviour was self-assessed using the revised 21-item Three-Factor Eating Questionnaire (TFEQ-R21), which comprises three domains: Uncontrolled Eating (UE, 6 items), which describes loss of control over food intake in response to hunger or external stimuli; Emotional Eating (EE, 6 items), which describes overeating during periods of negative mood; and Cognitive Restraint (CR, 9 items), which describes the conscious restriction of food intake to control body weight and shape [2–5]. The raw scores were converted to a scale from zero to 100, whereby higher values indicate greater levels of the respective eating behaviour. A validated cut-off score for excessive eating behaviour is presently lacking. However, a score of 50 was proposed in previous literature [18] and has been utilised in a subsequent study [6].

### Personality traits and psychogenic needs

The Cesarec-Marke Personality Schedule measures eleven psychogenic needs [10, 11, 19, 20]. The questionnaire comprises 165 yes-no items; each of the 11 psychogenic needs are represented by 15 questions, the meaning of which is described in Table 1. In addition, an acquiescence scale measures the propensity to respond affirmatively regardless of the question asked and is included as a control scale in regression models with psychogenic needs. The personality dimensions extraversion-introversion and neuroticism-stability were measured with the Eysenck Personality Inventory [14, 15]. The questionnaire includes 57 yes-no items, 24 of which measure neuroticism and a further 24 extraversion. Nine questions represent a control scale that assesses the tendency to respond with social desirability bias, often referred to as the “lie scale,” the term henceforth utilised in the current study.

**Table 1:** Description of the CMPS psychogenic needs.

Psychogenic need	Description
Achievement (ACH)	The need to achieve something important and difficult, to compete with and surpass the performance of others
Affiliation (AFF)	The need to form close emotional relationships, to always have someone to share your feelings with, to stick with friends and remain loyal to them
Aggression (AGG)	The need to avenge an offence, to be angry and destroy things, to mock others and make fun of them
Defence of status (DST)	The need to defend one’s status, to refrain from actions or behaviour in order to avoid failure, to be sensitive to the opinions of others
Guilt feelings (GUI)	The need to have high ethical standards and a strong sense of duty, to often examine and regret past actions and thoughts
Dominance (DOM)	The need to be the leader, to influence others more than to be influenced, to assert one’s own opinion when it conflicts with the opinion of others
Exhibition (EXH)	The need to be noticed and be at the centre of attention, to be witty, funny, and entertaining, to shock and amaze others
Autonomy (AUT)	The need to feel independent, to be indifferent to the opinions of others, to avoid responsibility, to have the urge to do things that are considered immoral
Nurturance (NUR)	The need to help and care for others, to be willing to lend or give to people in need
Order (ORD)	The need for organisation, planning and cleanliness, getting annoyed with people who are not tidy and punctual
Succorance (SUC)	The need to be cared for both emotionally and practically, to feel abandoned and excluded when alone

*CMPS* Cesarec-Marke Personality Schedule.

### Body mass index (BMI)

The physical examination included measurement of weight and height, and body mass index was calculated as weight/height^2^ (in units of kg/m^2^). BMI was further categorised into underweight (BMI < 18.5), overweight (BMI ≥ 25) and obesity (BMI ≥ 30).

### Potential confounders

The participants completed a self-report questionnaire whereby information on the highest level of education was obtained, which in turn was divided into university education and lower education. The women also indicated whether they were living with a partner and answered questions on previous and current weight-loss diets. All but one of the current dieters had also been dieters in the past, so three mutually exclusive categories were defined: never, only in the past, and current dieting regardless of previous dieting. Consumption of sweets was categorised as never, a few sweets/day, and consumption several times/day. Leisure time physical activity (LTPA) was assessed in four categories, sedentary lifestyle, moderate activity ≥four hours/week, regular training and competitive sports. Coffee consumption was reported as cups/day, regardless of type of coffee or preparation method. Current tobacco use distinguished any tobacco use (cigarette, cigar, pipe, e-cigarette, moist snuff) and no tobacco use. General wellbeing was answered as a validated question on a 7-step Likert scale, with higher scores indicating better well-being [6].

### Analytic sample

Exclusion criteria were missing values regarding BMI (*n* = 2), eating behaviour (*n* = 27), or personality scores (*n* = 54). Five women were excluded due to pregnancy, as this temporarily changes weight status and eating behaviour. The final analytic sample comprised observations from 485 women.

### Ethical approval

Approval for the study was granted by the Regional Ethical Committee Gothenburg (258-16). The study complies with the Declaration of Helsinki and written informed consent was obtained from all participants.

### Statistical analysis

The correlation between psychogenic needs and personality scores was assessed using Pearson correlation coefficients. Differences in mean values of psychogenic needs and personality scores by excessive eating behaviours was assessed using *t*-tests accounting for unequal variances in the two groups. We used multiple logistic regression with automatic variable selection to identify individual psychological scores and background factors associated with each type of excessive eating behaviour. Psychogenic needs and personality traits were standardised to permit the comparison of effect sizes given the different units of measurements; this was done by converting the variables into z-scores. The results were presented as odds ratio (OR) with 95% confidence interval. The area under the receiver operating characteristic curve (AUROC) was used to assess the performance of the model, where a value of 0.5 represents random prediction and the value of 1 represents classification with perfect accuracy. Multiple linear regression analyses were carried out to examine the associations between BMI and psychogenic needs, personality traits, eating behaviours, and potential confounders. As BMI had a right-skewed distribution the variable was log-transformed to ensure a normal distribution of residuals. The corresponding β-coefficients were transformed into (exp(β)-1) ×100%, which gives the percentage change in BMI when the independent variable is increased by one unit. Analyses of continuous BMI were supplemented by logistic regression of overweight and obesity, with variable selection from predictors as described above. Finally, cubic spline regression was used to illustrate the mutual associations between specific psychogenic needs, emotional eating, and BMI or the likelihood of overweight and obesity. Statistical analyses were performed using SAS v.9.4 and Matlab R2022a. The significance level was 0.05 (two-sided tests).

## Results

### Characteristics of the study population

The sample consisted of two age strata, women aged 38 (44%) and 50 (56%). The prevalence of overweight and obesity was given by 38% and 13%, respectively. About one-third of the women had a university education, and 79% of the women lived with a partner. Dieting was common in this sample: 70% of women had dieted or were currently dieting. Only eight percent said they led a sedentary lifestyle while 56% reported regular training or competitive sports. Eighteen percent of women currently consumed tobacco products. Mean values of scores describing eating behaviour, psychogenic needs, and Eysenck personality traits are shown in Supplementary Table S1.

### Correlations between psychogenic needs and Eysenck personality traits

The mutual correlations between individual psychogenic needs and personality traits are shown in Supplementary Table S2. Two sets with three variables each were identified, the need for defence of status, guilt feelings and neuroticism as well as the needs for aggression and exhibition and extraversion, that showed positive correlations within and negative correlations between sets (|r|> 0.4). The correlation structure is further illustrated in Supplementary Fig. S1.

### Associations between psychological factors and excessive eating behaviour

Women with excessive emotional eating scored higher on aggression, defence of status, guilt, succorance, acquiescence and neuroticism, and lower on extroversion and the Eysenck-lie scale compared to women without excessive emotional eating (Table 2). Several of these differences were also observed for excessive uncontrolled eating, albeit with smaller effect size. Women with excessive cognitive restraint showed higher scores for achievement, affiliation and nurturance compared to women without excessive cognitive restraint.

**Table 2:** Mean values (SD) of psychogenic needs, Eysenck Personality Inventory scores, and control variables by indicators of excessive eating behaviour^a^.

	Excessive uncontrolled eating		Excessive emotional eating		Excessive cognitive restraint	
	No (ref)	Yes	No (ref)	Yes	No (ref)	Yes
*N*	415	70	416	69	357	128
	Mean (SD)	Mean (SD)	Mean (SD)	Mean (SD)	Mean (SD)	Mean (SD)
Psychogenic needs						
Achievement	7.9 (2.9)	8.1 (2.9)	7.9 (2.9)	8.5 (2.7)	7.8 (3.0)	8.5 (2.7)**
Affiliation	8.9 (2.3)	9.5 (2.4)	9.1 (2.3)	8.7 (2.2)	8.9 (2.4)	9.4 (2.2)*
Aggression	5.2 (2.8)	6.0 (2.9)*	5.2 (2.8)	6.1 (2.9)*	5.4 (2.8)	5.3 (2.9)
Defence of status	6.0 (3.4)	7.4 (3.7)**	5.9 (3.4)	8.1 (3.8)***	6.2 (3.5)	6.2 (3.6)
Guilt feelings	6.4 (3.2)	7.4 (3.5)*	6.3 (3.1)	7.8 (3.8)**	6.4 (3.3)	6.7 (3.2)
Dominance	9.5 (3.3)	8.8 (3.4)	9.5 (3.3)	8.7 (3.7)	9.4 (3.4)	9.3 (3.1)
Exhibition	8.1 (3.2)	8.4 (3.5)	8.2 (3.2)	7.9 (3.6)	8.1 (3.3)	8.1 (3.1)
Autonomy	7.5 (2.2)	7.9 (2.3)	7.5 (2.2)	7.9 (2.4)	7.6 (2.3)	7.4 (2.1)
Nurturance	11.5 (2.3)	11.4 (2.8)	11.5 (2.4)	11.3 (2.6)	11.3 (2.5)	11.9 (2.2)*
Order	9.5 (3.2)	8.9 (2.9)	9.5 (3.1)	9.0 (3.3)	9.2 (3.2)	9.8 (2.8)
Succorance	8.7 (2.6)	9.1 (2.8)	8.6 (2.6)	9.4 (2.9)*	8.7 (2.7)	8.9 (2.5)
Acquiescence	44.6 (7.1)	47.8 (9.7)**	44.4 (7.4)	48.5 (8.0)***	44.8 (7.6)	45.5 (7.5)
Eysenck personality inventory						
Extraversion	14.0 (3.6)	13.5 (3.7)	14.1 (3.5)	12.9 (3.8)**	13.9 (3.7)	14.1 (3.3)
Neuroticism	7.8 (4.2)	9.9 (4.8)***	7.6 (4.2)	10.7 (4.6)***	8.2 (4.5)	7.9 (4.0)
Lie scale	3.0 (1.8)	2.3 (1.4)***	3.0 (1.8)	2.3 (1.4)***	2.9 (1.7)	3.1 (1.9)

*p < 0.05, **p < 0.01, ***p < 0.001.

^a^Independent sample *t*-test by excessive eating behaviour accounting for unequal variances if appropriate.

To determine which of the personality scores were independently associated with excessive eating behaviours, we performed logistic regression with automatic variable selection among psychological factors and potential confounders. Table 3 shows that the need to defend one’s status was associated with higher odds of excessive uncontrolled eating in a model including the Eysenck-lie scale and sweet consumption. The need for defence of status was also associated with excessive emotional eating, with one SD higher z-DST being associated with 61% higher odds for excessive emotional eating. The magnitude of the association with DST was greater than the equally positive association with neuroticism. Only psychogenic needs were selected in a model for excessive cognitive restraint, with the need for achievement being associated with higher odds, and the need for autonomy with lower odds of excessive cognitive restraint.

**Table 3:** Significant associations between excessive eating behaviours, psychogenic needs, and personality scores as well as potential confounders (OR with 95% CI)^a^.

	Excessive uncontrolled eating	Excessive emotional eating	Excessive cognitive restraint
Cases/total	70/483	69/483	127/480
University education			0.58 (0.37, 0.90)
Sweets (ref = never)			
Few sweets/day	5.79 (1.76, 19.1)	2.58 (1.04, 6.43)	0.42 (0.25, 0.71)
Several times/day	15.8 (3.96, 63.6)	14.0 (4.20, 46.6)	0.12 (0.04, 0.42)
Dieting (ref = never)			
Former only		3.77 (1.60, 8.92)	2.82 (1.55, 5.13)
Current		9.08 (3.21, 25.7)	13.0 (5.92, 28.6)
Psychogenic needs			
z-ACH			1.39 (1.09, 1.76)
z-AUT			0.78 (0.62, 0.97)
z-DST	1.44 (1.11, 1.86)	1.61 (1.18, 2.20)	
Eysenck Personality Inventory			
z-neuroticism		1.45 (1.06, 1.97)	
z-lie scale	0.65 (0.49, 0.88)	0.69 (0.50, 0.95)	
AUROC	0.73	0.80	0.77

*ACH* achievement, *AUT* autonomy, *DST* defence of status, *AUROC* area under the receiver operating characteristic curve.

^a^Logistic regression of excessive eating behaviour on personality scores as well as potential confounders (age, education, living with a partner, dieting behaviour, LTPA, consumption of sweets, consumption of coffee, tobacco use, and wellbeing) using automatic variable selection.

In sensitivity analyses we tested the associations of individual questions that form the respective psychogenic needs and the neuroticism scale. A positive answer to the question ‘do you often think about situations that have been embarrassing for you?’ more than doubled the odds for excessive uncontrolled eating, OR = 2.64 (1.53, 4.54), adjusted for sweet consumption and the Eysenck-lie scale (AUROC = 0.74). The same question was also associated with excessive emotional eating, OR = 2.18 (1.19, 3.99), as was the question ‘do you find it difficult to maintain your self-confidence when you are together with confident and superior people?’, OR = 3.01 (1.63, 5.56), adjusted for sweet consumption and dieting behaviour (AUROC = 0.81). No single item from the neuroticism scale was associated with excessive emotional eating in a model including the items of DST described above. Among questions for achievement and autonomy the individual question ’do you want to achieve something important?’ was most strongly associated with excessive cognitive restraint, OR = 2.33 (1.24, 4.37). In the same model, two questions contributing to the need for autonomy (in inverse direction), ‘do you have the feeling that your opinions and perceptions are almost always in line with what people in general think and feel?’ and ‘do you think that the person who goes his own way without taking into account prevailing opinions will sooner or later fail?’ were associated with the outcome, OR = 1.61 (1.00, 2.60) and OR = 1.79 (1.06, 2.99), respectively, adjusted for sweet consumption and dieting behaviour (AUROC = 0.76).

### Is the association between eating behaviour and BMI influenced by personality traits or psychogenic needs?

To characterise the association between eating behaviour and BMI, two analyses were conducted using both the continuous scores and the dichotomised versions representing excessive eating behaviour (Table 4). To assess the extent to which these associations were confounded by psychological factors, each model was run with and without adjustment for personality traits and psychogenic needs. We performed linear regression with backward variable selection among psychological factors and covariates, retaining the variables for eating behaviour.

**Table 4:** Associations between BMI and eating behaviour adjusted for selected covariates and personality scores (*n* = 482)^a^.

	Effect on BMI (%)				
	Basic model	+CMPS/EPI		Basic model	+CMPS/EPI
Eating behaviour			Eating behaviour		
z-EE	2.7 (0.9, 4.4)	2.9 (1.2, 4.7)	EE > 50	4.3 (0.2, 8.5)	5.3 (1.1, 9.6)
z-UE	−0.9 (−2.6, 0.8)	−0.8 (−2.4, 0.9)	UE > 50	−1.0 (−4.9, 3.0)	−0.8 (−4.6, 3.2)
z-CR	1.2 (−0.3, 2.7)	1.2 (−0.2, 2.7)	CR > 50	0.8 (−2.4, 4.0)	0.6 (−2.5, 3.8)
Covariates					
University education	−3.9 (−6.6, −1.1)	−3.5 (−6.2, −0.8)		−3.8 (−6.5, −1.0)	−3.5 (−6.2, −0.7)
Living with a partner	−5.2 (−8.2, −2.1)	−4.8 (−7.8, −1.7)		−5.4 (−8.4, −2.3)	−5.0 (−8.0, −1.9)
Dieting (ref = never)					
Former only	9.1 (5.7, 12.5)	8.7 (5.4, 12.0)		10.3 (7.1, 13.7)	10.0 (6.8, 13.3)
Current	16.2 (10.4, 22.3)	16.0 (10.2, 22.0)		19.1 (13.3, 25.1)	19.0 (13.3, 25.0)
LTPA (ref = sedentary)					
≤4 times/week	−3.6 (−8.4, 1.4)	−3.4 (−8.2, 1.5)		−3.3 (−8.1, 1.7)	−3.1 (−7.9, 1.9)
Regular training	−7.7 (−12.2, −3.0)	−7.5 (−12.0, −2.8)		−7.4 (−11.9, −2.6)	−7.2 (−11.7, −2.4)
Competitive sports	−15.1 (−20.3, −9.6)	−14.7 (−19.9, −9.2)		−15.2 (−20.4, −9.6)	−14.8 (−20.0, −9.3)
Psychogenic needs					
z-DST		−2.8 (−4.1, −1.4)			−2.7 (−4.1, −1.3)
z-ACQ		1.5 (0.1, 2.9)			1.5 (0.1, 2.9)
R_adj_^2^	0.25	0.27		0.23	0.25

*EE* emotional eating, *UE* uncontrolled eating, *CR* cognitive restraint, *LTPA* leisure time physical activity, *DST* defence of status, *ACQ* acquiescence.

^a^Linear regression of log-BMI on eating behaviours, and variable selection from the set of covariates (age, university education, living with a partner, LTPA, dieting, consumption of sweet consumption of coffee, tobacco use, and wellbeing) as well as psychogenic needs and Eysenck personality traits.

The results showed a general consistency in the associations between log-BMI and the continuous and dichotomised eating-behaviour scores. Using the continuous scores, participants were found to have 2.7% higher BMI values per SD of emotional eating in the basic model, and 2.9% higher BMI when further adjusted for psychological factors, while other domains of eating behaviour were not associated with BMI (left columns in Table 4). From the set of psychological factors, only defence of status acquiescence were selected. The inverse association between defence of status and BMI was also observed after excluding acquiescence, with an effect size of −2.3 (−3.6, −1.0) % per SD. Further adjustment for neuroticism showed that the inverse association between DST and BMI, −2.3 (−3.8, −0.9) %, was stronger than the equally inverse association with neuroticism, −1.5 (−3.1, 0.1) %.

**Fig. 1:**
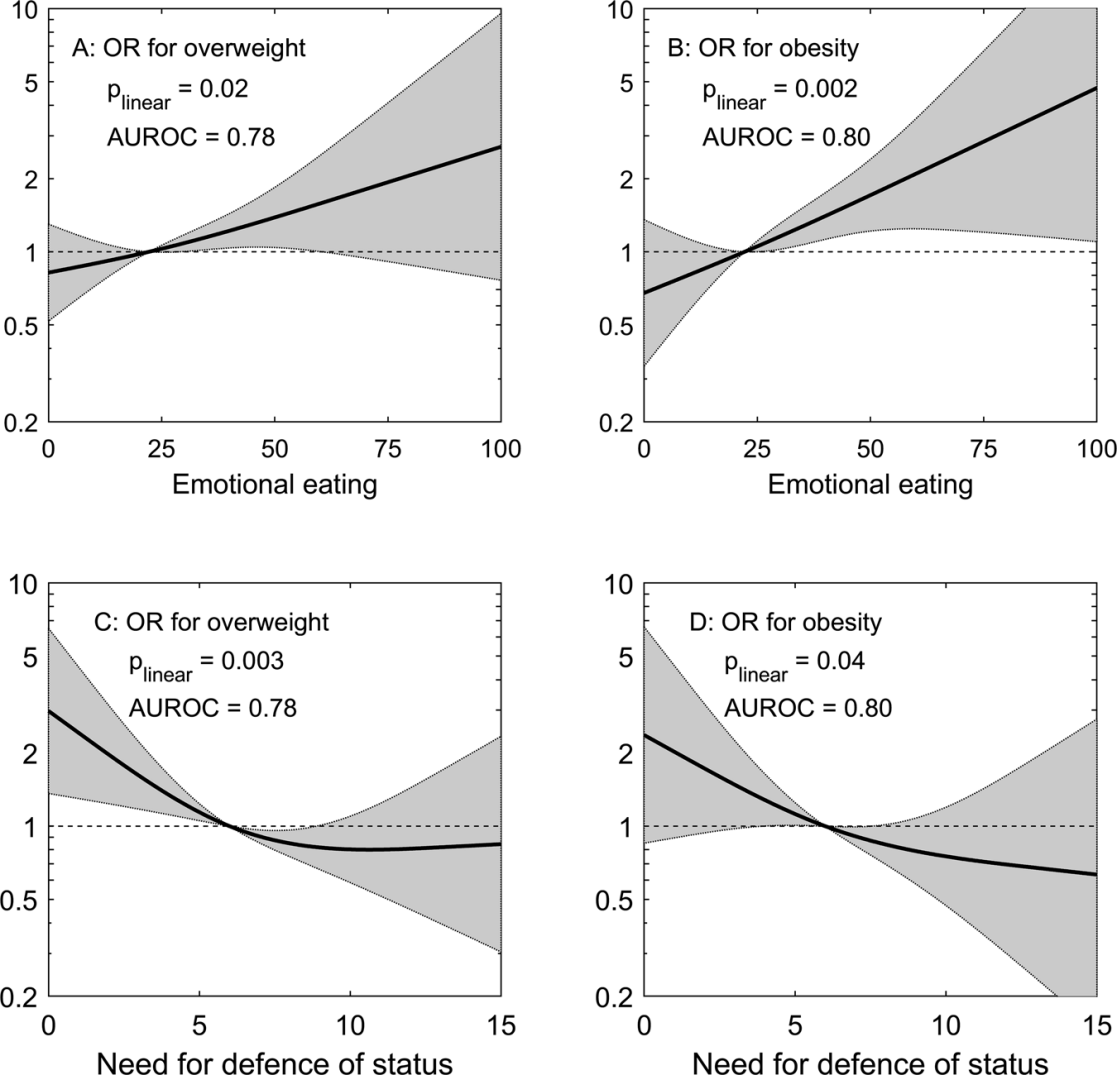
Emotional eating and the need for defence of status in relation to overweight and obesity. Spline regression for risk of overweight /obesity as a function of emotional eating behaviour (**A**, **B**) and defence of status (**C**, **D**)^**a**^. ^**a**^Logistic regression using restricted cubic splines (ref = median) in models including emotional eating (EE), need for defence of status (DST), living with a partner, dieting behaviour, and physical activity (LTPA). Only linear terms of EE and DST were statistically significant.

The same covariates were selected in models that contained excessive eating behaviours (right columns in Table 4). Excessive emotional eating was associated with 4.3% higher BMI compared to non-excessive eating behaviour, and this increased to 5.3% when adjusted for DST and ACQ. Overall, past and current dieting were associated with higher BMI, while higher education, living with a partner, and higher physical activity were associated with lower BMI. Positive associations with emotional eating and negative associations with DST in mutually adjusted regression models were also observed with respect to risk for overweight and obesity (Fig. 1).

Sensitivity analyses were performed to obtain a detailed picture of the association between DST and BMI. Spline regression showed an inverse J-shaped association for log-BMI as a function of DST but the positive association with BMI at the upper end of the DST spectrum was not statistically significant (Supplementary Fig. S2). The non-linear association between neuroticism and log-BMI was weaker than for DST and log-BMI (not shown).

## Discussion

### Summary of results

In this study of women aged 38 and 50, eating behaviour measured by the Three-Factor Eating Questionnaire was found to be associated with both psychogenic needs and personality traits. However, only defence of status was associated with excessive uncontrolled eating in mutually adjusted models, while defence of status and neuroticism were both positively associated with excessive emotional eating. Models for excessive cognitive restraint found a positive association with the need for achievement and a negative association with the need for autonomy. Regression models for body mass index confirmed that emotional but not uncontrolled or restrained eating behaviours was positively associated with BMI. The strength of the association between emotional eating and BMI was not reduced when further adjusted for psychological factors. Defence of status was most strongly associated with body weight and showed negative associations with BMI, overweight, and obesity despite its positive correlation with emotional eating behaviour. Independent associations of BMI with high emotional eating and low defence of status could indicate different psychological dispositions underlying excess body weight, which in turn require different therapeutic approaches.

### Associations between psychological factors and eating behaviour

The need to defend one’s status was the predominant correlate of both uncontrolled and emotional eating behaviours, offsetting associations with other psychological factors. The exception was the neuroticism scale that contributed to excessive emotional eating independently of DST. Neuroticism and the need for DST share aspects of self-consciousness and emotional instability but their correlation was only moderate. However, psychogenic needs in general, and the need for DST in particular almost always assess the quality of interactions with other people, whereas the Eysenck neuroticism scale assesses states of dysphoric mood irrespective of reason or cause. Consequently, specific problems such as rumination about embarrassing situations or low self-confidence in the presence of confident and superior people may be more directly related to overeating than mood and psychosomatic symptoms assessed with the neuroticism scale. The link between neuroticism and emotional eating has been observed before [7, 21–23], but the new finding that it is associated with the need for defence of status could help to identify and treat specific causes for over-eating.

Although cognitive restraint was not associated with body weight, it is interesting to note that a higher need for achievement and a lower need for autonomy were independently associated with restrained eating behaviour. While the need to master difficult tasks may include maintaining a healthy diet, a reduced need for autonomy could be a sign of sensitivity to the opinions and habits of others, which also promotes restrained eating behaviour. To our knowledge, this is the first time that associations between personality scores and restrained eating behaviour have been examined in a population-based sample that also took into account confounding factors such as dieting.

### Associations of psychological factors and eating behaviours with weight status

The results for the continuous scores of the Three-Factor Eating Questionnaire (TFEQ) in relation to BMI are consistent with our previous study [6], with minor differences due to fewer observations and covariates not included here. The main result was that emotional eating was the predominant eating behaviour associated with obesity in middle-aged women independent of uncontrolled or restrained eating and of confounders including dieting behaviour. The positive association between emotional eating and BMI is now confirmed in regression models adjusting for psychological factors of which the need to defend one’s status was the most important. Since DST correlated positively with emotional eating, one might have expected it to be at least partly responsible for the association between emotional eating and BMI. However, the opposite was observed: since *low* but not high DST was associated with higher BMI as well as overweight and obesity, including DST in the model for BMI or weight status strengthened the association with emotional eating. The latter is formally a result of negative confounding, as the associations of DST with emotional eating and BMI point in opposite directions. Consequently, high levels of emotional eating and low defence of status are independent risk factors and may account for different mechanisms behind the development of excess body weight. While emotional eating by definition and by association with elevated BMI may represent overeating in response to dysphoric mood states [23, 24], the mechanism underlying a low need for defence of status and body weight is less clear. It is possible that a low need for defence of status or low neuroticism is associated with a lower basal metabolic rate [25, 26], which favours sedentary behaviour and accumulation of excess weight.

The inverse association between DST and, to a lesser extent, neuroticism with BMI or obesity appears to contradict previous reports of positive associations between neuroticism and weight status in both sexes [27], and among women when sex-stratified analyses were presented [8, 28–30]. However, a meta-analysis of nine international cohort studies found no overall association between neuroticism as measured by the Five-factor model and the prevalence or incidence of obesity, but the heterogeneity between studies was large [31]. In a recent population study from Germany a positive association between neuroticism and obesity in covariate-adjusted models was observed in men but not in women [32]. In our study, there was a U-shaped association between the need for DST and BMI, with a weak positive association at high levels of DST (Supplementary Fig. S2) but only the inverse association was observed in confounder-adjusted models. The use of self-reported anthropometric data [27–29, 32] and not testing for nonlinearity or effect modification by gender may contribute to the discrepancy between the present and previous results, but differences in the content of personality measures may be even more important: Sutin and Terracciano [33] discuss that scales measuring the anger and impulsivity aspects of neuroticism tend to show positive associations with BMI [8], whereas scales assessing emotional vulnerability show more variability, including inverse associations [26]. The latter may well apply to our results, as the psychogenic need to defend one’s status is strongly associated with the vulnerability facet of neuroticism.

Interventions targeting underlying thought patterns rather than eating behaviours per se, such as cognitive behavioural therapy [34, 35] and mindfulness-based approaches [36], have been shown to be successful in reducing emotional eating and excess body weight. In contrast, patients with weight problems and low levels of neuroticism or need for defence of status, who do not display the typical signs of emotional eating, may benefit from more conventional methods of weight loss, including a healthier diet and a more physically active lifestyle. It is hypothesised that high self-confidence and low sensitivity to the thoughts of others protect these individuals from the pressure to conform to contemporary body image and dieting culture, and part of this association is mediated by a low basal metabolic rate [25, 26]. Supplementary Fig. S3 illustrates the proposed risk factors of obesity as well as their treatment.

### Strengths and limitations

The data in this study was taken from the Population Study of Women in Gothenburg, which consists of a relatively large sample of working-age women, with a high participation rate by today’s standards (68%). The objective assessment of anthropometry is a strength of the study that distinguishes it from other studies using self-reported weight and height, which carries the risk of under-reporting. The adjustment of regression models for the control variables ‘acquiescence’ and ‘the propensity to give socially desirable answers’ is another strength. One of the limitations of the study is that it only included female participants. While emotional eating behaviour is most strongly related with weight status in women, uncontrolled eating may be more decisive in men [37], and there may be gender differences with respect to the psychogenic needs as well. Furthermore, the study is cross-sectional, rendering it difficult to establish causality. Longitudinal studies examining weight development after the treatment of psychological problems, e.g. through cognitive behavioural therapy, could help to understand whether psychogenic needs give rise to excessive eating behaviour or vice versa.

## Conclusions

This study identified specific psychological factors underlying excessive eating behaviours, which both supports the psychogenic cause of overeating and gives directives for its treatment. For the middle-aged women studied here, the need to defend one’s status and aspects of neuroticism were shown to be associated with excessive emotional eating, a behaviour that also correlates with obesity. The highly specific questions underlying the assessment of psychogenic needs may inform the treatment of emotional eating and unhealthy body weight. The association between *low* need for defence of status and excessive body weight independent of emotional eating was a novel finding that illustrates the heterogeneity of risk factors for overweight and obesity. While not directly linked with eating behaviour it should be the subject of future studies.

## Supplementary information

Supplement

## Data Availability

The data sets used and analysed during the current study are available from the corresponding author upon reasonable request.
