# Supplementary material for: Personality, eating behaviour, and body weight: results from the population study of women in Gothenburg 2016/17
Source: Int J Obes (Lond). 2025 Apr 4;49(7):1272–9. doi: 10.1038/s41366-025-01764-y (PMC12283360; doi:10.1038/s41366-025-01764-y)

**Table S1:** Basic characteristics of women participating in the 2016/17 survey of the Population Study of Women in Gothenburg.

|  | Total ^a^ | Mean (SD) | Range |
| --- | --- | --- | --- |
| Three-factor Eating Questionnaire: |  |  |  |
| Emotional eating (EE) | 485 | 25.3 (24.1) | 0 – 100 |
| Uncontrolled eating (UE) |  | 27.9 (19.5) | 0 – 92.6 |
| Cognitive restraint (CR) |  | 37.2 (21.5) | 0 – 94.4 |
| Eysenck Personality Inventory: |  |  |  |
| Neuroticism | 485 | 8.1 (4.4) | 0 – 22 |
| Extraversion |  | 13.9 (3.6) | 3 – 23 |
| Social desirability scale (lie scale) |  | 2.9 (1.7) | 0 – 8 |
| Psychogenic needs: |  |  |  |
| Achievement (ACH) | 485 | 8.0 (2.9) | 0 – 14 |
| Affiliation (AFF) |  | 9.0 (2.3) | 1 – 15 |
| Aggression (AGG) |  | 5.3 (2.8) | 0 – 15 |
| Defence of status (DST) |  | 6.2 (3.5) | 0 – 15 |
| Guilt (GUI) |  | 6.5 (3.3) | 0 – 15 |
| Dominance (DOM) |  | 9.4 (3.3) | 0 – 15 |
| Exhibition (EXH) |  | 8.1 (3.2) | 0 – 15 |
| Autonomy (AUT) |  | 7.5 (2.2) | 1 – 14 |
| Nurturance (NUR) |  | 11.5 (2.4) | 3 – 15 |
| Order (ORD) |  | 9.4 (3.1) | 0 – 15 |
| Succorance (SUC) |  | 8.7 (2.6) | 2 – 14 |
| Acquiescence (ACQ) |  | 45.0 (7.6) | 24 – 79 |
| BMI (kg/m^2^) | 485 | 24.7 (4.5) | 16.5 – 49.1 |
| Wellbeing ^b^ | 485 | 3.7 (1.3) | 0 – 6 |
| Coffee (cups/day) | 485 | 2.6 (2.0) | 0 – 15 |
|  |  | N (%) |  |
| Excessive eating behavior: EE > 50 | 485 | 69 (14) |  |
| UE > 50 |  | 70 (14) |  |
| CR > 50 |  | 128 (26) |  |
| Overweight: BMI > 25 kg/m^2^ | 485 | 183 (38) |  |
| Obesity: BMI > 30 kg/m^2^ |  | 61 (13) |  |
| Age strata: 38 years | 485 | 215 (44) |  |
| 50 years |  | 270 (56) |  |
| University education | 482 | 331 (69) |  |
| Living with a partner | 485 | 384 (79) |  |
| Sweets: never | 483 | 97 (20) |  |
| Few sweets/day |  | 356 (74) |  |
| Several times/day |  | 30 (6) |  |
| Dieting: never | 485 | 145 (30) |  |
| Former only |  | 286 (59) |  |
| Current |  | 54 (11) |  |
| LTPA: sedentary | 485 | 39 (8) |  |
| Moderate |  | 172 (36) |  |
| Regular training |  | 230 (47) |  |
| Competitive sports |  | 44 (9) |  |
| Current tobacco use | 485 | 88 (18) |  |

^a^ total number of non-missing observations for each variable

^b^ seven categories, higher is better

**Table S2**: Correlation coefficients for psychogenic needs and personality traits (n = 485).

|  | AFF | AGG | DST | GUI | DOM | EXH | AUT |  | NUR | ORD | SUC | AQC | **Neuroticism** | **Extraversion** | Lie scale |
| --- | --- | --- | --- | --- | --- | --- | --- | --- | --- | --- | --- | --- | --- | --- | --- |
| ACH | 0.10* | 0.12** | 0.09* | 0.19*** | 0.27*** | 0.25*** | 0.20*** |  | 0.06 | 0.04 | -0.01 | 0.35*** | 0.09 | 0.14** | -0.05 |
| AFF | 1 | -0.03 | 0.01 | 0.07 | 0.06 | 0.13** | -0.19*** |  | 0.36*** | -0.04 | 0.35*** | 0.03 | 0.01 | 0.37*** | 0.12** |
| AGG |  | 1 | 0.11* | 0.10* | 0.15*** | 0.21*** | 0.33*** |  | -0.15** | -0.02 | 0.06 | 0.28*** | 0.29*** | -0.01 | -0.25*** |
| **DST** |  |  | 1 | **0.56***** | **-0.47***** | -0.32*** | -0.07 |  | -0.07 | 0.14** | 0.25*** | 0.36*** | **0.44***** | **-0.43***** | -0.04 |
| **GUI** |  |  |  | 1 | -0.36*** | -0.24*** | 0.02 |  | 0.07 | 0.13** | 0.20*** | **0.45***** | **0.55***** | -0.33*** | 0.06 |
| **DOM** |  |  |  |  | 1 | **0.56***** | 0.11* |  | 0.04 | -0.11* | -0.14** | -0.12** | -0.33*** | **0.45***** | -0.04 |
| **EXH** |  |  |  |  |  | 1 | 0.11* |  | 0.02 | -0.16*** | 0.03 | -0.07 | -0.14** | **0.51***** | -0.11* |
| AUT |  |  |  |  |  |  | 1 |  | -0.15** | -0.23*** | -0.18*** | 0.29*** | 0.24*** | -0.12** | -0.13** |
| NUR |  |  |  |  |  |  |  |  | 1 | -0.10* | 0.20*** | -0.01 | -0.03 | 0.28*** | 0.16*** |
| ORD |  |  |  |  |  |  |  |  |  | 1 | 0.09* | 0.02 | -0.05 | -0.18*** | 0.24*** |
| SUC |  |  |  |  |  |  |  |  |  |  | 1 | 0.04 | 0.26*** | 0.08 | -0.05 |
| AQC |  |  |  |  |  |  |  |  |  |  |  | 1 | 0.46*** | -0.17*** | -0.05 |
| Neuroticism |  |  |  |  |  |  |  |  |  |  |  |  | 1 | -0.32*** | -0.18*** |
| Extraversion |  |  |  |  |  |  |  |  |  |  |  |  |  | 1 | -0.01 |

* *p* < 0.05, ** *p* < 0.01, *** *p* < 0.001

Partial Pearson correlation coefficients adjusted for age, values with |r| > 0.4 in bold

ACH = achievement, AFF = affiliation, AGG = aggression, DST = defence of status, GUI = guilt, DOM = dominance, EXH = exhibition, AUT = autonomy, NUR = nurturance, ORD = order, SUC = succorance, ACQ = acquiescence.

**Figure S1**: Mutual correlations (|r| > 0.4) between psychogenic needs and acquiescence as well as Eysenck personality traits.


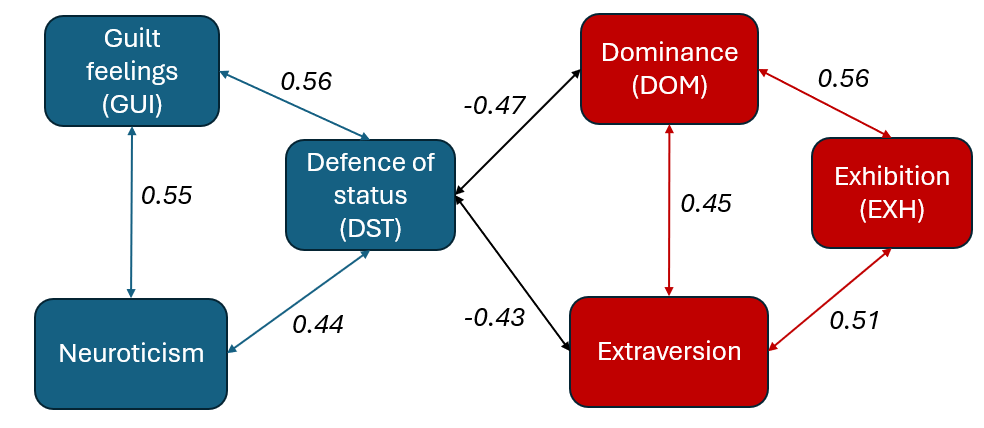


**Figure S2**: Illustration of cross-sectional associations between the need to defend one’s status, the score for emotional eating as well as the logarithm of body mass index (log BMI) from unadjusted restricted cubic spline regression.


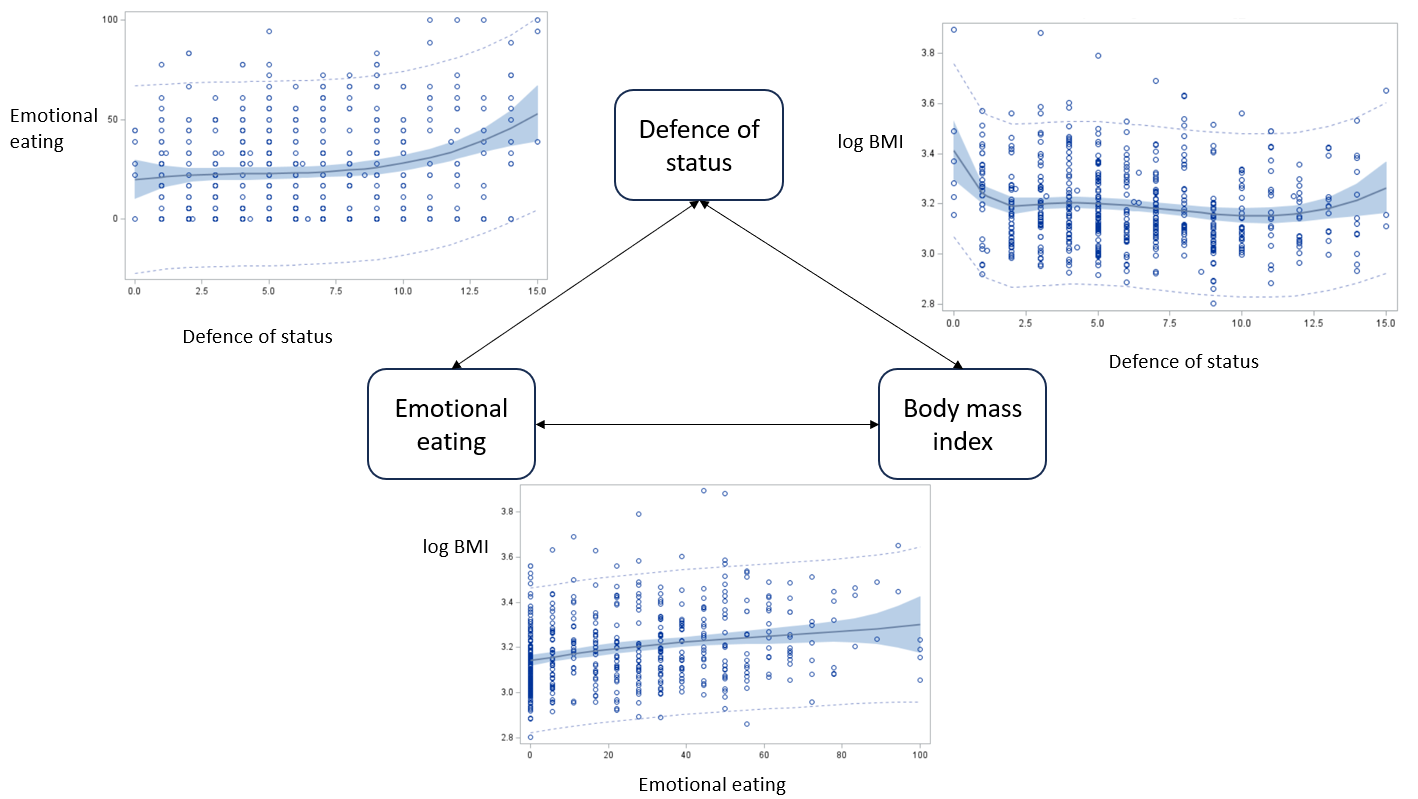


**Figure S3**: Illustration of possible pathways and treatments for obesity risk factors.


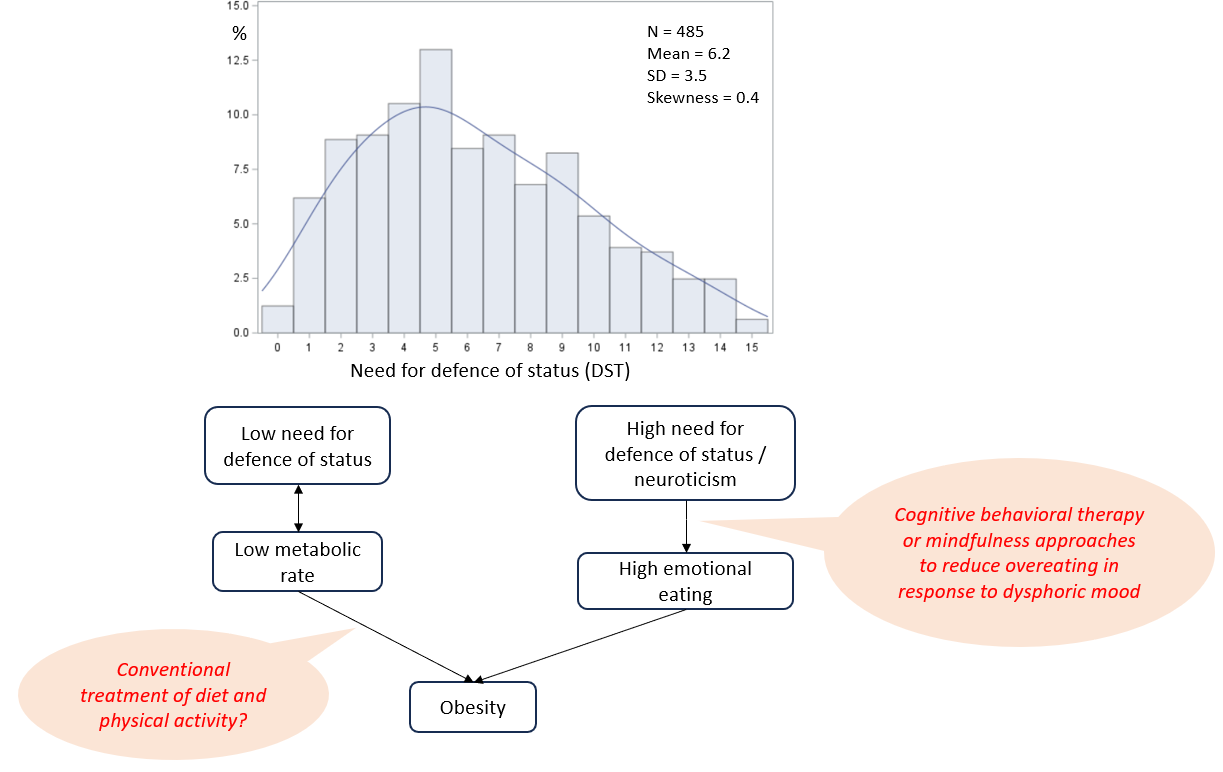

Supplement: Supplementary file 1 — Supplement [file 41366_2025_1764_MOESM1_ESM.docx]
